# The role of awareness, appreciation, and communication satisfaction in shaping employee engagement: evidence from the organizational health behavior index

**DOI:** 10.3389/fpsyg.2026.1671007

**Published:** 2026-02-09

**Authors:** Abad Alzuman, Muath Jaafari, Zaiba Ali, Rahila Ali, Lina Ibrahim Bakadam

**Affiliations:** 1 Department of Management, College of Business Administration, Princess Nourah Bint Abdulrahman University, Riyadh, Saudi Arabia; 2 Organizational Culture and Internal Communication, ICM, Amjad Watan, Riyadh, Saudi Arabia; 3 Faculty of Management, Jagran Lakecity University, Bhopal, India

**Keywords:** appreciation, awareness, communication satisfaction, employee engagement, moderation, organizational health behavior index (OHBI), Saudi Arabia, SEM

## Abstract

A healthy organization fosters an environment in which employees feel valued, motivated, and connected to their work. This study examines the relationship between organizational health and employee engagement using the Organizational Health Behavior Index (OHBI) as a multifaceted diagnostic framework, focusing on the joint effects of awareness, appreciation, and communication satisfaction. Data were collected from 7,548 employees across public, private, and semi-government sectors in Saudi Arabia, and Structural Equation Modeling (SEM) was employed to test direct and moderating relationships among the study constructs. Confirmatory Factor Analysis (CFA) demonstrated excellent model fit (CFI = 0.978; RMSEA = 0.048), with all factor loadings exceeding 0.70, supporting convergent and discriminant validity. The results indicate that awareness (*β* = 0.230), appreciation (*β* = 0.152), and communication satisfaction (*β* = 0.070) significantly predict employee engagement (*p* < 0.001). Moderation analysis further reveals that communication satisfaction strengthens the relationship between awareness and employee engagement (*β* = 0.082), while slightly attenuating the effect of appreciation (*β* = −0.027), highlighting its dual role as a contextual moderator. The findings offer actionable insights for enhancing employee engagement and fostering healthier organizational environments across diverse sectors. Although the study is situated within the Saudi Arabian context, the OHBI demonstrates broad applicability across different countries, industries, and organizational settings, providing a practical framework for assessing organizational health and supporting human resource management interventions

## 1 Introduction

In an era marked by rapid economic transformation and workforce modernization, employee engagement has emerged as a central concern for both scholars and practitioners. Extensive research demonstrates that engaged employees contribute to superior organizational performance, enhanced innovation, increased job satisfaction, and stronger organizational citizenship behaviors (Bakker and Albrecht, 2018; Bailey et al., 2017). Within the context of Saudi Arabia’s Vision 2030—which emphasizes economic diversification, human capital development, and public sector efficiency—understanding the drivers of employee engagement has become particularly critical (Alyamani, 2025; Aziz, n.d.). Organizational reforms under Saudi Vision 2030 emphasize human capital development and employee engagement as strategic priorities for long-term institutional sustainability (Yamin, 2019).

Although traditional antecedents of engagement, such as leadership, compensation, and job design, have been widely examined (Kahn, 1990; Lai et al., 2020), recent scholarship has increasingly highlighted the importance of organizational health practices in cultivating psychologically safe and empowering work environments. Organizational health, defined as an organization’s capacity to align people, culture, and strategy to sustain long-term performance, is now widely recognized as a precursor to employee engagement and well-being (Deepalakshmi et al., 2024; Bakker and Albrecht, 2018). In this regard, employee awareness, communication satisfaction, and appreciation-related behaviors play a crucial role in fostering trust, motivation, and engagement within organizations (Asiedu-Appiah and Zoogah, 2019; Baqir et al., 2020; Sun et al., 2023; Verčič and Špoljarić, 2020).

The Organizational Health Behavior Index (OHBI) was created to put these aspects into action. It is a multi-dimensional diagnostic instrument that includes eight essential health drivers: awareness, appreciation, relations, communication satisfaction, engagement, employee voice, persona, and organizational culture. Based on strategic and humanistic ideas, OHBI gives a complete view of how to improve organizational health, employee engagement, and organizational culture that is in line with both local policy needs and global standards (Jaafari et al., 2023). Governments, the commercial sector, nonprofit groups, and academia have created several assessment and diagnostic frameworks to improve organizational health and performance. A framework that integrates both qualitative and quantitative evaluation methodologies is necessary to address all critical aspects of organizational health and behavior. Consequently, the OHBI scale was divided into two subscales: “Subscale A” for quantitative assessments and “Subscale B” for qualitative evaluations. The integration of the two methodologies can enhance the credibility, validity, and applicability of this study, while also advancing the domain of organizational health behavior research. The OHBI can help create an environment at work that boosts productivity, engagement, and retention. Organizations can compare their health behavior ratings to industry standards or best practices using the index.

Although the Organizational Health Behavior Index (OHBI) has demonstrated robust psychometric properties in prior studies (Jaafari et al., 2023), the interplay among its dimensions and their influence on employee engagement—particularly in emerging economies such as Saudi Arabia—remains underexplored. Furthermore, while communication is widely acknowledged as a catalyst for effective organizational functioning, its moderating role in shaping the relationships between awareness, appreciation, and employee engagement warrants further empirical validation, which is addressed in the present study.

Accordingly, this study aims to address these gaps by examining how OHBI dimensions—specifically awareness and appreciation—affect employee engagement both directly and indirectly, with communication satisfaction conceptualized as a strategic moderator. Using a large and diverse sample of 7,548 employees from multiple sectors across Saudi Arabia and employing structural equation modeling (SEM), this study provides timely evidence on how organizational health behaviors align with national objectives of building a productive, engaged, and resilient workforce under Vision 2030 (Soleiman and Ming, 2025).

In light of the aforementioned theoretical and empirical gaps, this study is guided by the following research questions:

1. RQ1: To what extent does awareness significantly predict employee engagement within the context of Saudi organizations?
2. RQ2: How does appreciation contribute to the variance in employee engagement across diverse institutional sectors?
3. RQ3: What is the direct relationship between communication satisfaction and employee engagement, and to what degree does it act as an independent correlate of engagement??
4. RQ4: Does communication satisfaction significantly moderate the relationship between awareness and employee engagement, thereby amplifying or attenuating this relationship?
5. RQ5: Does communication satisfaction exert a moderating influence on the association between appreciation and employee engagement, and if so, in what direction?

## 2 Literature review

### 2.1 Organizational health and employee engagement

Organizational health is increasingly recognized as a critical factor influencing employee engagement and performance outcomes (Merdrousians, 2024). In any organization, organizational health plays a paramount role in employee well-being. Organizations must strive to provide employees with a healthy work environment so they can perform their roles to the best of their abilities. Camp et al. (2024) define organizational health as the extent to which organizational leaders effectively “run the place,” relating to decision-making, resource allocation, daily operations, and team leadership. It mainly comprises three elements: (1) how the organization rallies behind a shared vision and strategy, (2) how effectively it executes corporate strategy, and (3) how effectively it reinvents itself over time (De Smet and Mygatt, 2021). In the context of public sector transformations—such as Saudi Arabia’s Vision 2030—the need to understand and enhance organizational health is paramount for aligning employee behavior with strategic goals (PwC Middle East, 2025).

The literature explains that organizational health is essential for improving employee engagement because it influences dimensions vital to engagement, such as alignment, clear communication and workflow, well-being and development, organizational fairness, meaningful work, and innovation (Whatfix, 2025). Employees who feel valued and appreciated by their organization are more likely to be committed to their work because they perceive their contributions as meaningful and valuable to the organization (Merdrousians, 2024). Recent empirical studies in the Saudi context highlight the growing importance of organizational practices that foster employee engagement and well-being during periods of institutional transformation (Alharthi et al., 2023).

### 2.2 Awareness and employee engagement

Employee awareness refers to individuals’ understanding of their organization’s goals, policies, and expectations. Awareness strengthens employees’ sense of purpose and alignment, thereby enhancing intrinsic motivation (Alabdaly and Almayali, 2021). Ghorooneh and Malekinia (2015) defined organizational health in terms of characteristics and specifications such as communication, perceptions of organizational strategies and politics, employee competence, manpower capability, management skills, employee morale, the workplace, employees’ awareness of the organization’s mission, cooperation, demographic characteristics of the workforce and education, and professional improvement and development, all of which influence employee behavior. Highlighting its significance, Usman and Musa (2012) stated that employees who possess high levels of organizational awareness are able to accomplish tasks more efficiently, understand organizational processes, make fewer errors arising from misunderstandings of organizational structure, and behave appropriately within the organization. Employees’ awareness of the organizational management structure is crucial for establishing rapid and optimized decision-making processes. As a result, this significantly reinforces and accelerates overall organizational business management (Khuen and Dileep, 2015).

### 2.3 Appreciation and employee engagement

Recognition and appreciation, which can also be referred to as intrinsic means of motivating employees, have become essential organizational phenomena. When it comes to fostering intrinsic satisfaction among employees, recognition and appreciation serve as important tools for managers, organizations, industrial counselors, and HR professionals in promoting employee motivation and organizational success (Abdullah et al., 2016).

In essence, consistent appreciation is a powerful mechanism through which organizations can cultivate a positive work environment, strengthen employee commitment, and encourage discretionary effort, ultimately leading to improved performance and organizational success (Suhail et al., 2025). In collectivist cultural contexts—such as Saudi Arabia—appreciation further supports social cohesion and interpersonal harmony (Alshammari et al., 2023).

Rai et al. (2018) stated that rewards and recognition function both as mechanisms for fulfilling standard job requirements and as incentives that encourage employees to go beyond formal role expectations through greater involvement in the workplace. Rewards may take various forms, including monetary compensation, durable goods, or non-monetary benefits, and serve as expressions of appreciation for employees’ contributions (Hasibuan, 2019). In addition, recognition is a key organizational practice for enhancing employee morale. According to Chevalier et al. (2022), recognition refers to organizational or managerial efforts aimed at making employees feel valued and appreciated as exemplary contributors through their actions. In the current organizational context, career opportunities aligned with goal congruence contribute directly to strategic fit, creating key drivers of employee engagement through motivational mechanisms such as rewards and recognition, which reflect the ambitions and aspirations of the workforce committed to organizational growth and sustainable progress (Khurape and Punse, 2019). Recognizing and appreciating employees’ contributions through effective communication channels can further foster a supportive and motivating work environment (Enyan et al., 2023).

### 2.4 Communication satisfaction and employee engagement

Engaging communication practices, such as active listening, open dialogue, and transparent information sharing, foster employee engagement by creating a sense of empowerment, recognition, and ownership (Bakker et al., 2011). Additionally, communication that provides timely feedback, guidance, and support enhances employees’ knowledge, skills, and motivation, ultimately leading to higher job performance and job satisfaction (Chen et al., 2017). Communication that provides timely feedback, guidance, and support further enhances employees’ knowledge, skills, and motivation, contributing to higher performance levels (Bakker et al., 2011). Effective communication improves job satisfaction, commitment, employee engagement, and performance. Communication channels that facilitate timely and relevant information flow contribute to employees’ sense of connectedness, satisfaction, and engagement (Gilbert et al., 2018).

Research indicates that effective internal communication significantly enhances employee engagement by fostering transparency and trust (Sun et al., 2023; Yue et al., 2021). Qin and Men (2023) further found that internal communication has a substantial influence on employee motivation by promoting a sense of trust and inclusion within the organization. Consequently, this improves job satisfaction by establishing a favorable work environment in which employees feel appreciated and motivated to contribute to the achievement of organizational goals. Prior research has shown that internal communication plays a critical role in shaping employee trust, engagement, and voice within organizations (Macmillan and Men, 2021).

#### 2.4.1 Theoretical framework

The Resource-Based View (RBV) and High-Performance Work Systems (HPWS) form the theoretical basis of this research by conceptualizing awareness, appreciation, and communication satisfaction as strategic organizational resources and HR practices that amplify employees’ capabilities, motivation, and opportunities to perform. Awareness reflects access to strategic information and goal clarity, aligning with RBV and the Ability–Motivation–Opportunity (AMO) framework through the concept of line of sight, whereby employees understand their roles and how they contribute to organizational outcomes. Appreciation represents motivation-enhancing practices that convey value and recognition, whereas communication satisfaction constitutes opportunity-enhancing activities that provide voice, participation, and positive social exchange within the organization.

From a Social Exchange Theory perspective, these resources foster high-quality reciprocal relationships, as employees who are informed, recognized, and engaged through equitable and transparent communication are more likely to reciprocate with higher levels of engagement. According to Self-Determination Theory (SDT), awareness fulfills needs for competence and meaningfulness, appreciation satisfies needs for relatedness and acknowledgment, and communication satisfaction fulfills needs for autonomy and voice. Collectively, these need-satisfaction processes explain why the three OHBI dimensions simultaneously predict employee engagement. Within this resource- and interaction-based model, communication satisfaction is conceptualized not only as a resource that directly influences other resources but also as a contextual factor that amplifies—or attenuates—the motivational benefits of awareness and appreciation, thereby providing theoretical justification for its hypothesized moderating role.

Building on the theoretical foundations and empirical evidence presented in the literature, it is evident that organizational health factors such as awareness and appreciation play a vital role in shaping employee engagement. Furthermore, communication satisfaction has emerged as a critical moderator that may strengthen or alter these relationships. Based on this research questions, the current study proposes the following hypotheses to examine the direct and interactive relationships of these variables with employee engagement.

*H1:* Awareness is positively associated with employee engagement.

*H2:* Appreciation is positively associated with employee engagement.

*H3:* Communication Satisfaction is positively associated with employee engagement.

*H4:* Communication Satisfaction moderates the relationship between awareness and employee engagement.

*H5:* Communication Satisfaction moderates the relationship between appreciation and employee engagement.

The structural model for this study is based on theories that seek to explain how the Organizational health components like awareness, appreciation and communication satisfaction are associated with employee engagement in Saudi organizations.

## 3 Research methodology

### 3.1 Study design

This research employed a quantitative research design with a cross-sectional, survey-based approach to examine the hypothesized relationships among variables. The study followed a two-stage structural equation modeling approach, incorporating confirmatory factor analysis (CFA) to validate the measurement model and test the hypothesized structural relationships. The rationale for adopting a quantitative strategy lies in its capacity to provide statistical evidence, test theoretical constructs, and enhance the generalizability of findings (Ishtiaq, 2019). The study aimed to examine the associations between OHBI sub-dimensions—awareness, appreciation, and communication satisfaction—and employee engagement, drawing on strategic HRM perspectives such as the Resource-Based View (RBV) (Lubis, 2022) and High-Performance Work Systems (HPWS) frameworks (Peethambaran and Naim, 2025). The methodology involved a large-scale national survey, robust psychometric testing, and advanced multivariate analyses using SEM and CFA. Table 1 outlines the sequence of studies, sample groups, and analytical steps implemented across the phases of the research process.

**Table 1:** Summary of analytical steps.

Stage	Analytical focus	Sample group	Methods/analyses
Stage 1.1	Instrument design and data collection	*n* = 7,548	Questionnaire developed based on OHBI (Subscale A); purposive and convenience sampling across 13 Saudi regions
Stage 1.2	Descriptive analysis	Same sample	Descriptive statistics (mean, SD, skewness, kurtosis) to test data normality and response trends (Table 4)
Stage 1.3	Reliability testing	Same sample	Internal consistency measured using Cronbach’s alpha for each construct and overall scale (Tables 5, 6)
Stage 1.4	Inter-item correlation and construct distinctiveness	Same sample	Pearson correlation matrix used to assess internal item convergence and early discriminant validity (Table 7)
Stage 1.5	Measurement model evaluation (CFA)	Same sample	Confirmatory Factor Analysis (CFA); evaluation of model fit indices, factor loadings, CR, and AVE (Tables 8–10; Figure 2)
Stage 1.6	Discriminant validity confirmation	Same sample	Fornell–Larcker Criterion and HTMT Ratio used to verify construct distinctiveness (Tables 11, 12)
Stage 2.1	Structural model testing and hypothesis validation	Same sample	Structural Equation Modeling (SEM); testing direct associations of awareness, appreciation, and communication satisfaction (Table 13)
Stage 2.2	Moderation analysis	Same sample	Interaction terms (Awareness × Communication Satisfaction, Appreciation × Communication Satisfaction) tested via SEM (Table 14; Figure 3)

### 3.2 Instrument and conceptual framework

**Figure 1:**
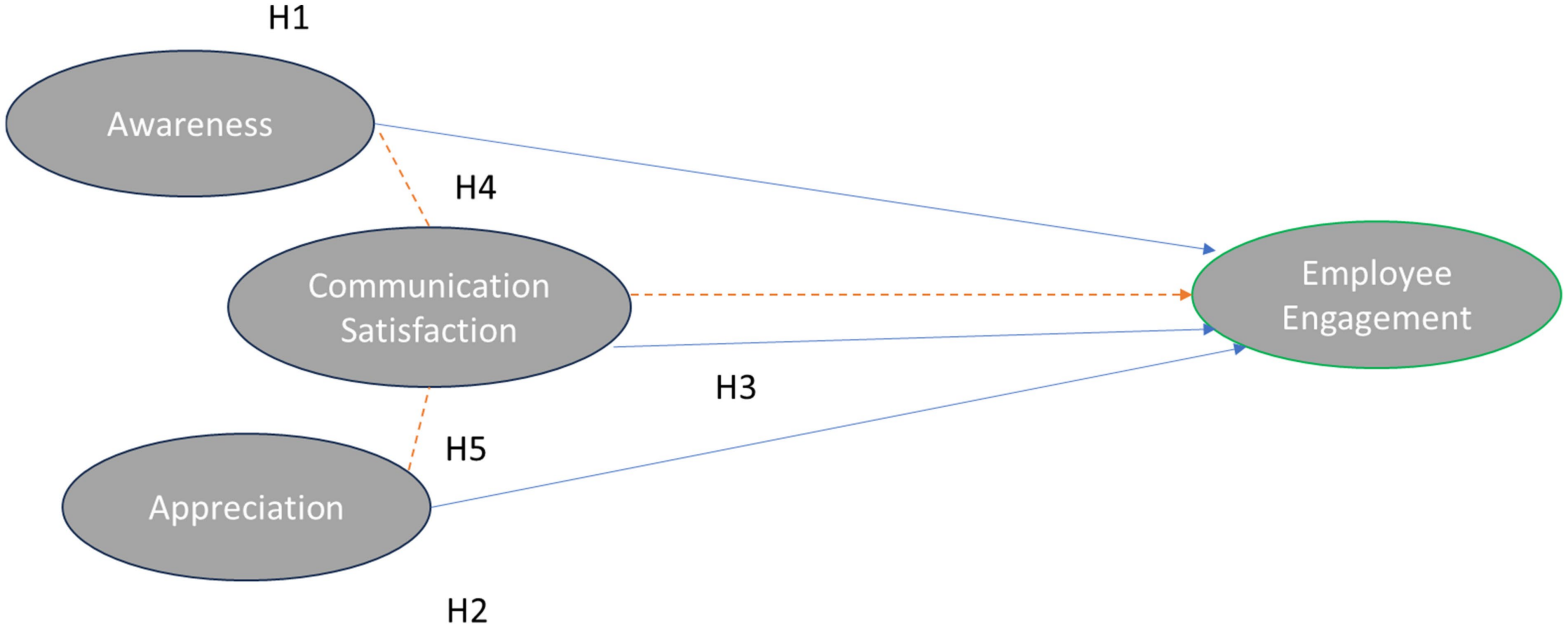
Conceptual model based on OHBI integrates both direct and moderating associations to assess the interplay between organizational health dimensions and employee engagement.

This study employed a cross-sectional survey design to examine the relationships among organizational awareness, appreciation, communication satisfaction, and employee engagement in the context of Saudi Arabian workplaces (Kothari, 2019) (see Figure 1). Data were collected using the Organizational Health Behavior Index (OHBI), a validated, multidimensional instrument that integrates both quantitative and qualitative measures of organizational health. The OHBI definitions for the dimensions considered in the current study are as follows.

Awareness is the foundational layer of organizational health and refers to employees’ understanding of organizational strategy, values, policies, rights, and internal communications. It captures the extent to which individuals are informed about their role in the organization’s vision and how aligned they feel with the broader direction of the organization.

Appreciation refers to the mutual exchange of recognition and respect between leaders, employees, and the organization. It reflects how consistently employees feel valued for their contributions through both formal and informal recognition mechanisms.

Communication Satisfaction in the OHBI focuses on employees’ perceptions of the quality, effectiveness, and accessibility of internal communication channels. It encompasses satisfaction with the activation of internal communication activities, perceived reach and inclusiveness, and the overall effectiveness of communication processes.

Employee Engagement reflects the depth of psychological and emotional involvement employees exhibit in their work, team relationships, and connection to the organization. It is measured through indicators such as engagement with work, engagement with colleagues, alignment with the corporate vision, and the employee Net Promoter Score (eNPS).

Data were collected using the Organizational Health Behavior Index (OHBI), a scale designed to measure organizational health across any type of organization based on three levels of needs (basic needs, corporate needs, and strategic needs) and eight factors: awareness, relations, appreciation, communication satisfaction, engagement, employee persona, employee voice, and organizational culture. Together, these factors contribute to a healthy organizational culture, excellence in performance, and the achievement of strategic objectives. The OHBI provides an accessible yet effective roadmap for leaders and managers to promote organizational health (see Appendix A). The scale contains no reverse-scored items.

Subscale A of the OHBI is divided into five dimensions: awareness (four items), appreciation (three items), employee engagement (three items), relations (three items), and communication satisfaction (three items). Scores for each dimension are calculated by summing responses to the respective items. Similarly, the total score for Subscale A is obtained by summing responses across all 17 items, yielding a possible range from 17 to 85. A normalized score above the normalized mean indicates good organizational health and behavior, whereas a lower score reflects poor organizational health and behavior.

The OHBI model provides a diagnostic framework that enables organizations to assess internal behavioral patterns, evaluate systematic performance, and implement targeted interventions to enhance organizational health and employee engagement by identifying strengths and weaknesses across these dimensions. The OHBI supports both public and private institutions in achieving operational excellence and fulfilling strategic objectives. For the present study, only the quantitative component of the OHBI (Subscale A) was used in the analysis. Subscale A includes 17 items representing the five selected dimensions: Awareness, Appreciation, Relations, Communication Satisfaction, and Employee Engagement. Each item was rated on a 5-point Likert scale ranging from 1 (Strongly Disagree) to 5 (Strongly Agree).

The Relations dimension was originally included in the OHBI conceptual framework but was excluded from the present study based on empirical and theoretical considerations. During the scale refinement process, Relations items demonstrated unstable factor loadings and cross-loadings with Appreciation and Communication Satisfaction, suggesting that interpersonal dynamics did not emerge as a distinct latent construct in this context. Including this dimension resulted in poorer overall model fit and violations of discriminant validity; retaining it would have compromised the psychometric robustness of the measurement model. From a theoretical perspective, the primary objective of this study was to examine the direct and moderating effects of organizational awareness, appreciation, and communication satisfaction on employee engagement. These dimensions were selected as they represent core strategic and behavioral principles central to organizational transformation and employee-focused strategies in Saudi organizations aligned with Vision 2030. In addition, although the OHBI includes a qualitative subscale (Subscale B) capturing employee voice and contextual narratives, this component was excluded from the analysis due to its open-ended nature and incompatibility with structural equation modeling (SEM).

Table 2 presents the items included in Subscale A and their thematic categorization.

**Table 2:** OHBI Subscale ‘A’ items and dimensions taken into the current study model.

S.no	Item	Theme
1	I am aware of my organization’s vision, mission, and goals.	Awareness
2	I am implementing my organization’s values in my daily work.	Awareness
3	I am aware of my organization’s external and internal news.	Awareness
4	I am aware of my rights and privileges as an employee in my organization.	Awareness
5	The appreciation my line manager shows for my work and accomplishments is satisfactory to me.	Appreciation
6	My company supports my efforts by providing the resources required to accomplish my tasks more effectively.	Appreciation
7	I rarely think about leaving my organization to work someplace else.	Appreciation
8	I am keen on promoting my organization’s accomplishments and activities, both on my personal social media profiles and during social events.	Employee engagement
9	During work hours, time flies by without me noticing as I focus on my tasks and responsibilities.	Employee engagement
10	I am familiar with the professional backgrounds of my colleagues in the same department, their health conditions, and how to interact with them.	Employee engagement
11	I would recommend my organization as a great place to work for my colleagues and family.	Employee engagement
12	My organization interacts positively with global awareness days, standing out compared to other organizations.	Communication satisfaction
13	I am satisfied with the communication services offered by my organization last year (emails, programs, activities).	Communication satisfaction
14	Communication Satisfaction was able to reach me through various communication channels.	Communication satisfaction

### 3.3 Sampling and data collection

The data were collected via an online, self-administered questionnaire hosted on the ICM.ai platform, an AI-based employee engagement and experience solution commonly adopted by Saudi organizations. Data collection was conducted from July to December 2024. A total of 7,986 employees initiated the survey, and 7,548 responses were retained in the final sample after data cleaning, resulting in a usable response rate of 94.5%. The survey link was distributed through internal communication channels and professional networks to employees working in public, private, and semi-government organizations across all 13 administrative regions of the Kingdom of Saudi Arabia. Of the 7,986 initiated responses, 438 were excluded following data screening. Responses were removed if they contained excessive missing data on key constructs (i.e., more than 20% of items missing on OHBI Subscale A), showed evidence of straight-lining (e.g., identical responses across all Likert-scale items), or contained illogical or inconsistent demographic information. This screening process ensured completeness and consistency of the data, resulting in a final sample of 7,548 employees.

A purposive convenience sampling approach was employed to intentionally target a diverse group of employees across sectors aligned with the national reform agenda under Vision 2030 (Stratton, 2023). Strategic sectors such as healthcare, aviation, public administration, and finance were prioritized due to their central role in national transformation and workforce modernization. Convenience sampling was further utilized to facilitate access to respondents through professional networks and the ICM.ai platform, enabling the collection of data from a wide range of geographical and organizational contexts. Although this approach yielded a large and heterogeneous sample, it does not permit statistical generalization to the entire Saudi workforce. Accordingly, potential response bias and limited generalizability should be considered when interpreting the findings, as respondents may systematically differ from non-respondents in terms of engagement levels or familiarity with organizational processes.

Nonetheless, the purposive element of the sampling strategy ensured alignment with the conceptual objectives of the study by focusing on industries most affected by Vision 2030 reforms. The convenience component allowed access to employees with firsthand knowledge of daily organizational operations, thereby enhancing the practical relevance of the data for model development. Such a combined sampling approach is appropriate for both exploratory and confirmatory research, particularly in studies involving diagnostic instruments applied in organizational practice, such as the Organizational Health Behavior Index (OHBI) (Stratton, 2023). This methodological approach aligns with national development priorities by enabling targeted assessment of workforce engagement and internal capacity development within priority sectors (Abubakar et al., 2024).

### 3.4 Data analysis procedure

Data were analyzed using IBM SPSS and AMOS version 26 following a two-stage structural equation modeling (SEM) approach. In the first stage, the measurement model was assessed using descriptive statistics, inter-item correlation analysis, and internal consistency checks through Cronbach’s alpha. Normality was evaluated using skewness and kurtosis values, all of which fell within acceptable thresholds (|skew| < 2; |kurtosis| < 7), indicating suitability for maximum likelihood estimation (Byrne, 2016). Linearity checks conducted through scatterplots revealed no evidence of curvilinear relationships.

Multicollinearity diagnostics indicated no concerns, with all variance inflation factor (VIF) values below 3.0, well under the commonly accepted cutoff of 5.0 (Hair et al., 2019). Independence of observations was ensured, as each participant completed the survey only once and no nested sampling structure was present. Confirmatory factor analysis (CFA) was then conducted to evaluate factor loadings, construct reliability, and convergent and discriminant validity. In the second stage, the structural model was tested to examine the direct associations of awareness, appreciation, and communication satisfaction with employee engagement. Additionally, moderation analysis was conducted by introducing interaction terms (Awareness × Communication Satisfaction and Appreciation × Communication Satisfaction) to assess whether communication satisfaction moderated the relationships between the independent variables and employee engagement. In the retained sample, item-level missing data were minimal and were handled using full information maximum likelihood (FIML) estimation in AMOS for both the CFA and SEM analyses. FIML utilizes all available data under the assumption that data are missing at random and is recommended when the proportion of missing data in latent variable models is low (Enders, 2010). Prior to conducting SEM, key model assumptions were tested to ensure analytical rigor. The large sample size (*N* = 7,548) substantially exceeded the recommended minimum for SEM, which typically ranges from 200 to 500 cases depending on model complexity (Hair et al., 2019; Kline, 2016).

#### 3.4.1 Assumption testing

Normality was evaluated using skewness and kurtosis statistics for all observed indicators. Although several variables exhibited moderate negative skewness and one item (AW2) showed a relatively higher deviation (skew = −1.468; kurtosis = 2.005), these values remain well within acceptable thresholds for structural equation modeling using maximum likelihood estimation, which tolerates skewness values up to |2| and kurtosis values up to |7| (Byrne, 2016). The distributional characteristics therefore do not indicate severe non-normality, particularly given the large sample size, and the assumption of normality was considered adequately met for model estimation.

### 3.5 Research significance

The Organizational Health Behavior Index (OHBI) is a novel tool that contributes to the fields of organizational behavior and human resource management by supporting improvements in organizational culture and employee engagement. This integrated methodological approach enabled the validation and application of the OHBI framework using a large and diverse sample, offering insights into how key organizational health constructs interact to influence employee engagement. The findings provide actionable knowledge for leaders and policymakers seeking to enhance workplace effectiveness, particularly within organizations operating under the evolving framework of Saudi Arabia’s Vision 2030 (Alnajim, 2024). The validated OHBI framework can serve as a practical guide for monitoring organizational transformation, strengthening employee engagement and organizational health, and driving sustainable performance across strategic sectors. Consequently, this research advances both theoretical understanding and practical interventions in organizational health, offering a scalable model applicable to other Gulf Cooperation Council (GCC) and emerging economies undergoing similar socio-economic transitions (Nassar, 2025).

## 4 Data analysis

### 4.1 Participant characteristics

The Organizational Health Behavior Index (OHBI) is a novel tool that can help improve organizational culture and employee engagement by contributing to the study of organizational behavior and human resource management. This integrated methodological approach enabled the validation and application of the OHBI framework in a large and diverse sample, offering insights into how key organizational health constructs interact to influence employee engagement. The findings provide actionable knowledge for leaders and policymakers seeking to enhance workplace effectiveness, particularly in organizations operating within the evolving framework of Saudi Arabia’s Vision 2030 (Alnajim, 2024). The validated OHBI framework can serve as a practical guide for monitoring organizational transformation, strengthening employee engagement and organizational health, and driving sustainable performance across strategic sectors. Thus, this research advances both theoretical understanding and practical interventions in organizational health, offering a scalable model for other Gulf Cooperation Council (GCC) and emerging economies undergoing similar socio-economic transitions (Nassar, 2025) (see Table 3).

**Table 3:** Participant characteristics (*n* = 7,548).

Variable	Category	f	%
Gender	Female	1,118	14.8%
	Male	6,430	85.2%
Location	Riyadh	5,200	68.9%
	Al-Bahah	55	0.7%
	Al-Jawf	112	1.5%
	Al-Qassim	160	2.1%
	Asir	68	0.9%
	Eastern	349	4.6%
	Hail	77	1.0%
	Jazan	99	1.3%
	Mecca	940	12.5%
	Medina	71	0.9%
	Northern Borders	233	3.1%
	Tabuk	97	1.3%
	Najran	87	1.2%
Industry	Agriculture	96	1.3%
	Airline	1,942	25.7%
	Art & Culture	135	1.8%
	Construction	330	4.4%
	Event Management	25	0.3%
	F&B	47	0.6%
	Finance	126	1.7%
	Funds	1,148	15.2%
	Government Affairs	305	4.0%
	Healthcare	1,623	21.5%
	Insurance	106	1.4%
	IPO	155	2.1%
	Military	440	5.8%
	Oil & Gas	201	2.7%
	Real Estate	344	4.6%
	Tech	10	0.1%
	Telecom	515	6.8%
Industry Type	Private	1,834	24.3%
	Government	4,876	64.6%
	Semi-Government	838	11.1%
Tenure	More than 48 months	5,906	78.2%
	24–48 months	1,131	15.0%
	12–24 months	96	1.3%
	6–12 months	232	3.1%
	3–6 months	118	1.6%
	Less than 3 months	65	0.9%
Position	Advisor	157	2.1%
	Manager	886	11.7%
	Director	738	9.8%
	Executive	152	2.0%
	Specialist	5,072	67.2%
	Team Lead	543	7.2%

### 4.2 Descriptive statistics

Table 4 presents the descriptive statistics, showing that all variables have mean scores above 3.7, indicating generally favorable responses from participants. Skewness values are negative, suggesting left-skewed distributions, which are common in perception-based studies where respondents tend to report positive agreement. Kurtosis values largely fall within ±2, confirming normality of distribution in line with established guidelines (Cain et al., 2017). The standard deviations indicate moderate variability across items. These results support the suitability of the data for further multivariate analyses, including Confirmatory Factor Analysis (CFA) and Structural Equation Modeling (SEM) (Marsh et al., 2019). Given that all items were measured on a 5-point Likert scale ranging from 1 (Strongly Disagree) to 5 (Strongly Agree), the observed left-skewed distributions suggest that the majority of respondents reported higher levels of agreement across the study constructs, reflecting generally favorable perceptions of organizational awareness, appreciation, communication satisfaction, and employee engagement.

**Table 4:** Descriptive statistics.

Item	Min	Max	Mean	Std. Dev.	Skewness	Kurtosis
AW1 (Awareness)	1.00	5.00	4.03	1.11	−1.132	0.609
AW2 (Awareness)	1.00	5.00	4.25	0.96	−1.468	2.005
AW3 (Awareness)	1.00	5.00	3.85	1.11	−0.870	0.140
AW4 (Awareness)	1.00	5.00	3.68	1.29	−0.728	−0.547
AP1 (Appreciation)	1.00	5.00	3.98	1.158	−1.159	0.597
AP2 (Appreciation)	1.00	5.00	3.96	1.205	−1.095	0.286
AP3 (Appreciation)	1.00	5.00	3.76	1.192	−0.844	−0.114
EE1 (Engagement)	1.00	5.00	3.97	1.06	−1.029	0.567
EE2 (Engagement)	1.00	5.00	3.94	1.093	−1.026	0.525
EE3 (Engagement)	1.00	5.00	3.92	1.124	−0.959	0.220
EE4 (Engagement)	1.00	5.00	3.91	1.134	−1.012	0.456
CS1 (Comm. Satisfaction)	1.00	5.00	4.04	1.05	−1.119	0.810
CS2 (Comm. Satisfaction)	1.00	5.00	3.99	1.07	−1.035	0.520
CS3 (Comm. Satisfaction)	1.00	5.00	3.94	1.04	−0.959	0.500

### 4.3 Reliability testing

#### 4.3.1 Reliability testing of variables

Table 5 presents the reliability scores for each construct. All Cronbach’s alpha values meet or exceed the acceptable threshold of 0.70 (Cronbach, 1951), confirming strong internal consistency across the dimensions of the OHBI model. These results indicate that each construct reliably measures its underlying concept and is suitable for subsequent confirmatory factor analysis and structural equation modeling.

**Table 5:** Reliability scores by construct.

Construct	Number of items	Cronbach’s alpha
Awareness (AW1, AW2, AW3, AW4)	4	0.841
Appreciation (AP1, AP2, AP3)	3	0.844
Employee engagement (EE1, EE2, EE3, EE4)	4	0.707
Communication Satisfaction (CS1, CS2, CS3)	3	0.851

#### 4.3.2 Internal consistency

Table 6 presents the inter-item correlation matrix, demonstrating strong internal consistency within each construct. Items related to Awareness (AW1–AW4), Appreciation (AP1–AP3), Employee Engagement (EE1–EE4), and Communication Satisfaction (CS1–CS3) exhibit moderate to high inter-correlations (mostly above 0.70 within their respective groups), indicating good convergent validity (Hair et al., 2010; Fornell and Larcker, 1981a, 1981b). Correlations between items from different constructs are considerably lower (generally below 0.45), supporting discriminant validity and confirming that the constructs are statistically distinct from one another (Henseler et al., 2015).

**Table 6:** Item inter-correlated matrix (Pearson).

Item	AW1	AW2	AW3	AW4	AP1	AP2	AP3	EE1	EE2	EE3	EE4	CS1	CS2	CS3
AW1	1.000	0.634	0.617	0.590	0.413	0.433	0.439	0.238	0.233	0.246	0.266	0.403	0.365	0.338
AW2		1.000	0.557	0.502	0.361	0.421	0.410	0.210	0.213	0.206	0.224	0.375	0.326	0.309
AW3			1.000	0.572	0.405	0.414	0.412	0.238	0.232	0.260	0.264	0.409	0.382	0.348
AW4				1.000	0.466	0.495	0.495	0.239	0.245	0.273	0.295	0.424	0.424	0.380
AP1					1.000	0.615	0.622	0.180	0.148	0.182	0.177	0.342	0.343	0.319
AP2						1.000	0.674	0.176	0.179	0.153	0.198	0.378	0.354	0.332
AP3							1.000	0.204	0.212	0.241	0.245	0.435	0.423	0.389
EE1								1.000	0.613	0.736	0.609	0.206	0.153	0.112
EE2									1.000	0.639	0.603	0.231	0.164	0.133
EE3										1.000	0.657	0.240	0.211	0.150
EE4											1.000	0.259	0.208	0.156
CS1												1.000	0.687	0.590
CS2													1.000	0.689
CS3														1.000

### 4.4 Measurement model analysis

#### 4.4.1 Confirmatory factor analysis (CFA)

**Figure 2:**
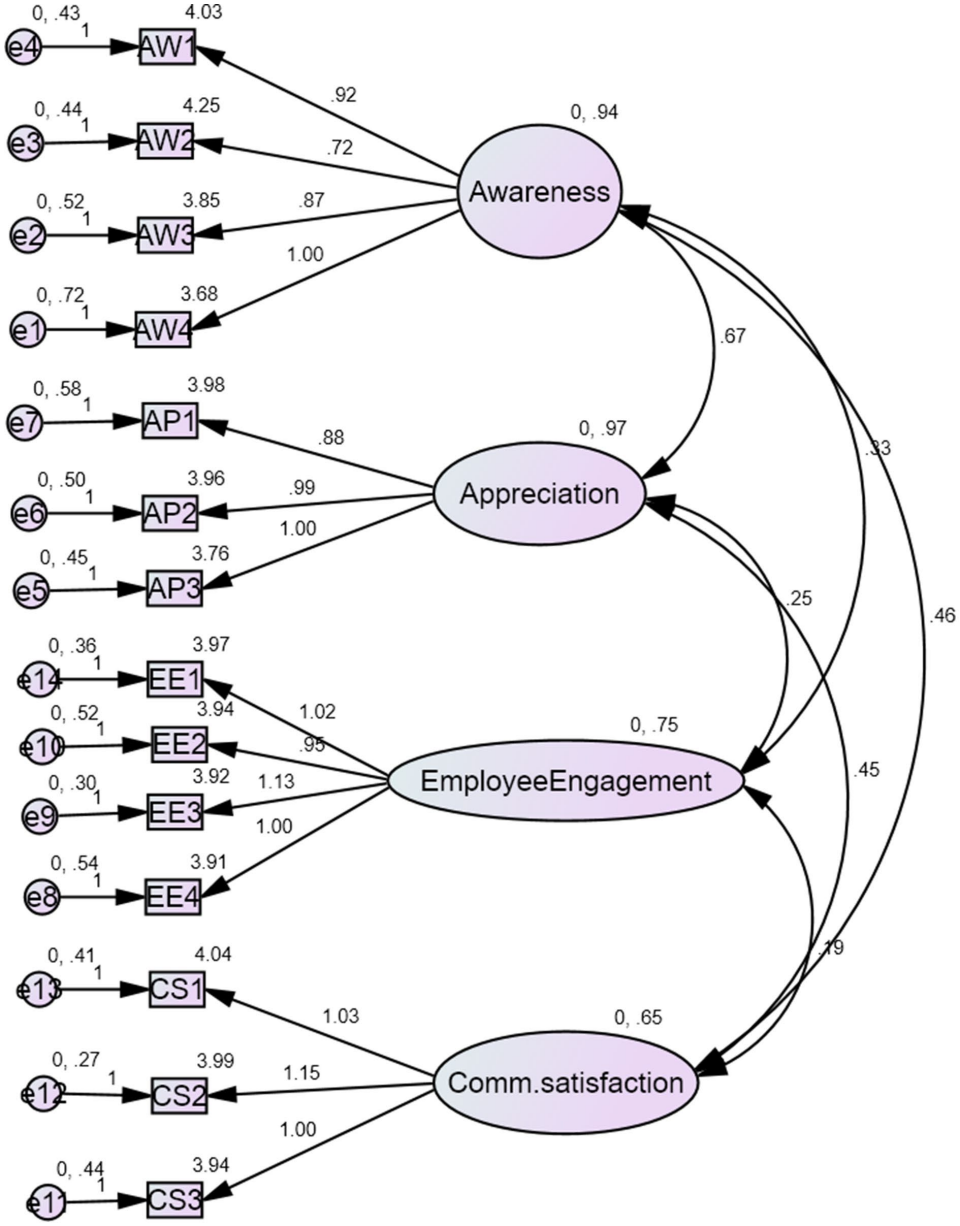
Confirmatory factor analysis (CFA) model illustrating the measurement structure of awareness (AW1–AW4), appreciation (AP1–AP3), employee engagement (EE1–EE4), and communication satisfaction (CS1–CS3) created on AMOS.V 26. Standardized factor loadings are presented along the arrows; all paths are statistically significant at *p* < 0.001. All coefficients are standardized estimates. EE, Employee Engagement; CS, Communication Satisfaction; AP, Appreciation; AW, Awareness.

To validate the measurement structure of the Organizational Health Behavior Index (OHBI), Confirmatory Factor Analysis (CFA) was conducted on a four-factor model comprising Awareness, Appreciation, Communication Satisfaction, and Employee Engagement. The results demonstrate excellent model fit, as shown in Figure 2 and Table 7. Key fit indices fall well within recommended thresholds (Hair et al., 2010), with values of CFI = 0.978, TLI = 0.967, and RMSEA = 0.048, supporting the adequacy of the measurement model. All standardized factor loadings were statistically significant (*p* < 0.001), ranging from 0.728 to 0.871, indicating strong convergent validity of the observed variables on their respective latent constructs (Cheung et al., 2024) (see Table 8).

**Table 7:** CFA model fit summary.

Model	χ^2^	χ^2^/df	df	NFI	RFI	IFI	TLI	CFI	RMSEA
Four factor model	1301.317	18.32	71	0.976	0.965	0.978	0.967	0.978	0.048

**Table 8:** Standardized factor loadings.

Construct	Item	Standardized loading
Awareness	AW1	0.807
	AW2	0.728
	AW3	0.761
	AW4	0.752
Appreciation	AP1	0.753
	AP2	0.809
	AP3	0.833
Employee engagement	EE1	0.827
	EE2	0.748
	EE3	0.871
	EE4	0.762
Communication satisfaction	CS1	0.792
	CS2	0.871
	CS3	0.773

The standardized factor loadings for all items were high and significant, indicating that the observed indicators were strong representations of their corresponding latent constructs. Factor loadings reflect the strength of the relationship between an item and its latent construct; values above 0.70 suggest that the item explains a substantial proportion of variance in the underlying construct and are commonly interpreted as evidence of convergent validity (Hair et al., 2019). The high loadings for Awareness, Appreciation, Communication Satisfaction, and Employee Engagement indicate that the items measuring these constructs are reliable and theoretically consistent.

The model showed strong fit with the following indices.

#### 4.4.2 Convergent and discriminant validity

Composite reliability (CR) and average variance extracted (AVE) values for each construct exceeded the recommended thresholds (CR > 0.70; AVE > 0.50), confirming construct reliability and convergent validity (Hair et al., 2010) (see Table 9). Discriminant validity was further established using the Fornell–Larcker criterion and the heterotrait–monotrait (HTMT) ratio. As shown in Table 10, all diagonal values (√AVE) exceeded the corresponding inter-construct correlations. Additionally, all HTMT values presented in Table 11 were below the recommended threshold of 0.85, providing further support for discriminant validity (Fornell and Larcker, 1981a, 1981b).

**Table 9:** Composite reliability and AVE.

Construct	Composite reliability (CR)	Average variance extracted (AVE)
Awareness	0.844	0.572
Appreciation	0.837	0.625
Employee engagement	0.879	0.634
Communication satisfaction	0.857	0.662

**Table 10:** Discriminant validity—Fornell-Larcker criterion.

Construct	AW	APP	EE	CS
Awareness	**0.756**			
Appreciation	0.702	**0.791**		
Employee engagement	0.389	0.296	**0.796**	
Comm. satisfaction	0.594	0.566	0.278	**0.814**

Note: Values on the diagonal (in bold) represent the square root of the Average Variance Extracted (√AVE) for each construct.

**Table 11:** Discriminant validity—HTMT ratio.

Construct	AW	APP	EE	CS
Awareness	–	0.791	0.475	0.701
Appreciation		–	0.405	0.639
Employee engagement			–	0.328
Comm. satisfaction				**–**

#### 4.4.3 Common method bias

Harman’s single-factor test was conducted to assess the potential presence of common method variance. All items measuring Awareness, Appreciation, Communication Satisfaction, and Employee Engagement were subjected to an unrotated principal component analysis. The results indicated that four factors with eigenvalues greater than 1.0 were extracted, and the first factor accounted for 40.77% of the total variance, which is below the recommended threshold of 50%. Therefore, no single factor accounted for the majority of the variance, suggesting that common method bias is unlikely to significantly affect the study findings (Podsakoff et al., 2003) (see Table 12).

**Table 12:** Total variance explained by principal component analysis (Harman’s single-factor test).

Component	Initial eigenvalues	Initial eigenvalues	Initial eigenvalues	Extraction sums of squared loadings	Extraction sums of squared loadings	Extraction sums of squared loadings
	Total	% of Variance	Cumulative %	Total	% of Variance	Cumulative %
1	5.708	40.774	40.774	5.708	40.774	40.774
2	2.311	16.504	57.279	2.311	16.504	57.279
3	1.270	9.075	66.353	1.270	9.075	66.353
4	1.008	7.201	73.554	1.008	7.201	73.554
5	0.498	3.555	77.109	**–**	**–**	**–**
6	0.446	3.183	80.292	**–**	**–**	**–**
7	0.408	2.912	83.205	**–**	**–**	**–**
8	0.401	2.866	86.070	**–**	**–**	**–**
9	0.384	2.740	88.810	**–**	**–**	**–**
10	0.374	2.673	91.484	**–**	**–**	**–**
11	0.342	2.443	93.926	**–**	**–**	**–**
12	0.331	2.365	96.291	**–**	**–**	**–**
13	0.276	1.969	98.260	**–**	**–**	**–**
14	0.244	1.740	100.000	**–**	**–**	**–**

### 4.5 Structural model analysis

Model fit was evaluated using multiple indices to determine whether the hypothesized structural model adequately represented the observed data. The χ^2^/df ratio is commonly used to assess overall model fit, with values below 3 indicating acceptable fit, although this index can be sensitive to large sample sizes (Kline, 2016). Incremental fit indices, including the Comparative Fit Index (CFI) and Tucker–Lewis Index (TLI), assess improvement over a baseline model, with values of 0.90 or higher indicating good fit (Hair et al., 2019). The Root Mean Square Error of Approximation (RMSEA) evaluates the degree of approximation error per degree of freedom, where values ≤ 0.08 indicate reasonable fit and values ≤ 0.06 reflect close fit (Byrne, 2016). The Standardized Root Mean Square Residual (SRMR) measures the average standardized residuals, with values ≤ 0.08 considered acceptable (Kline, 2016).

The standardized path coefficients for Awareness (*β* = 0.230, *p* < 0.001), Appreciation (*β* = 0.152, *p* < 0.001), and Communication Satisfaction (*β* = 0.070, *p* = 0.012) were all statistically significant, indicating meaningful positive effects on Employee Engagement. The model explained 41% of the variance in Employee Engagement (R^2^ = 0.41), demonstrating moderate explanatory power consistent with SEM standards. The final structural model exhibited strong fit across all indices (χ^2^/df = 3.10; CFI = 0.994; TLI = 0.988; RMSEA = 0.025), all of which fall well within recommended thresholds, indicating that the four-factor framework effectively captures the relationships among the core variables examined in this study.

The substantial improvement in model fit from the initial CFA (χ^2^/df = 18.32) to the final structural model (χ^2^/df = 3.10) can be attributed to anticipated modifications in both the measurement and structural models rather than to any irregularities in model estimation. The initial CFA represented an unconstrained item-level model that included several weaker indicators and no correlated error terms. Following recommended SEM procedures (Kline, 2016), the measurement model was refined by removing low-loading items and introducing theoretically justified within-construct error covariances. The theoretical structural model was subsequently tested using the refined measurement model, and the inclusion of theory-driven structural paths resulted in a more parsimonious model, which generally improves fit indices. Thus, the observed improvement reflects appropriate measurement refinement and theory-guided model specification (see Table 13).

**Table 13:** Model fit summary.

Model	χ^2^	χ^2^/df	df	NFI	RFI	IFI	TLI	CFI	RMSEA
Four Factor Model	9.569	3.1	3	0.991	0.983	0.994	0.988	0.994	0.025

As shown in Table 14, the structural paths revealed significant and positive associations between the three primary predictors—Awareness, Appreciation, and Communication Satisfaction—and Employee Engagement. Awareness (H1) exerted the strongest influence (*β* = 0.230), suggesting that when employees clearly understand their organization’s goals, values, and direction, their engagement is meaningfully enhanced. Appreciation also demonstrated a significant positive effect (*β* = 0.152), indicating that recognition and feeling valued contribute to higher engagement. Communication Satisfaction (H3) showed a smaller yet meaningful effect (*β* = 0.070), underscoring the importance of open, timely, and transparent communication in fostering an engaged workforce.

**Table 14:** Standardized path estimates and significance.

Hypothesis	Statement	Path coefficient (*β*)	Critical ratio (CR)	*p*-value	Supported
H1	Awareness → Employee Engagement	0.230	21.568	***	Yes
H2	Appreciation → Employee Engagement	0.152	14.267	***	Yes
H3	Communication Satisfaction → Employee Engagement	0.070	6.555	***	Yes
H4	Awareness × Communication Satisfaction → Employee Engagement (Moderation)	0.082	9.968	***	Yes
H5	Appreciation × Communication Satisfaction → Employee Engagement (Moderation)	−0.027	−3.232	0.001	No

Note: *** indicates statistical significance at *p* < 0.001.

The standardized path coefficients (*β*) represent both the direction and magnitude of the relationships among latent constructs. A positive coefficient indicates that increases in the predictor variable are associated with corresponding increases in the outcome variable, while the magnitude of the coefficient reflects the strength of the relationship (Byrne, 2016). Statistically significant coefficients (*p* < 0.05) indicate that these effects are unlikely to have occurred by chance and support the hypothesized structural pathways (Schumacker and Lomax, 2016). Collectively, these results demonstrate that Awareness, Appreciation, and Communication Satisfaction each play a meaningful role in shaping Employee Engagement within the proposed model.

**Figure 3:**
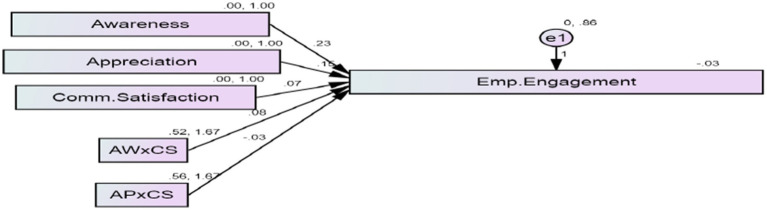
Path diagram of the structural model showing the direct relationships of awareness, appreciation, and communication satisfaction with employee engagement, as well as the moderating role of communication satisfaction in the relationships between awareness, appreciation, and engagement, via interaction terms (AWXCS and APXCS). AWXCS = Awareness × Communication Satisfaction; APXCS = Appreciation × Communication Satisfaction.

The model further examined the interplay between Communication Satisfaction and the other two factors. The findings indicate that Communication Satisfaction strengthens the positive relationship between awareness and employee engagement (*β* = 0.082). When organizational communication is effective, the positive association between employee awareness and engagement becomes more pronounced. However, H5 was not supported, as the interaction between Appreciation and Communication Satisfaction showed a small negative association with engagement (*β* = −0.027). In certain contexts, an excessive emphasis on recognition and communication may be perceived as overextended or lacking authenticity, potentially leading to communication fatigue or mixed signals. These findings highlight that communication functions not only as a direct predictor of engagement but also as a contextual factor that can strengthen—or, in some cases, weaken—the relationships between other organizational practices and employee engagement. Figure 3 presents a visual representation of these direct and moderating relationships.

### 4.6 Model equation

The interaction relationships were modeled using the following equation:

$$\begin{array}{l} \mathrm{EE}=\beta 0+\beta 1\cdot \mathrm{AW}+\beta 2\cdot \mathrm{AP}+\beta 3\cdot \mathrm{CS}\\ +\beta 4\cdot (\mathrm{AW}\times \mathrm{CS})+\beta 5\cdot (\mathrm{AP}\times \mathrm{CS})+\varepsilon \end{array}$$

where:

- EE = Employee Engagement (Dependent Variable)
- AW = Awareness (Independent Variable)
- AP = Appreciation (Independent Variable)
- CS = Communication Satisfaction (Moderator)
- AW×CS and AP×CSAP = interaction terms representing moderation relations
- ε = error term.

The model evaluates both direct relationships (*β*1, *β*2, *β*3) and moderation relationships (*β*4, *β*5) simultaneously.

## 5 Result and discussion

Managing a company’s human resources represents a foundational component of organizational management systems. The strategic significance of human resource management (HRM) in Saudi Arabia is closely linked to workforce composition and interpersonal relations within organizations. Previous research highlights the methods utilized in Saudi Arabia to improve governance through HRM practices, as well as the long-term strategies required for these practices to be effective. Abdaker et al. (2019) identified a lack of Middle Eastern empirical research examining how organizational policies and practices influence diversity management, retention strategies, incentive structures, and employee welfare schemes across public and private sectors. These findings underscore the importance of strategic HRM models, such as the SHRM–performance linkage, in promoting organizational health and employee well-being. Building on this foundation, the present study applies concepts from organizational behavior (OB) and HRM by examining how awareness, appreciation, and communication satisfaction—the three dimensions of the Organizational Health Behavior Index (OHBI)—influence employee engagement, a key predictor of organizational performance and sustainable workforce outcomes. This approach aligns with social exchange theory (Ahmad et al., 2023) and employee engagement models, including flow theory (Csikszentmihalyi, 2020) and Deloitte’s engagement model (Deloitte, 2025), which posit that employees’ workplace experiences are closely linked to engagement levels.

The study’s diverse sample of 7,548 employees—predominantly male (85.2%) and largely based in Riyadh (68.9%)—across public (64.6%), private (24.3%), and semi-governmental (11.1%) sectors enhances the generalizability of the findings. The predominance of long-tenured employees (78.2% with more than 48 months of experience) further strengthens the reliability of perceptions related to internal organizational practices. By integrating HRM and organizational behavior perspectives with the OHBI framework, this study contributes to a deeper understanding of how organizational health practices support employee engagement and organizational effectiveness in Saudi Arabia.

Table 4 shows consistently positive perceptions across all items, with mean scores ranging from 3.68 to 4.25. Skewness and kurtosis values were within acceptable limits, indicating that the data met assumptions of normality and were suitable for multivariate analysis. The scale demonstrated strong reliability, with an overall Cronbach’s alpha of 0.886 (Table 5), and all individual constructs—Awareness, Appreciation, Employee Engagement, and Communication Satisfaction—exceeding the 0.70 threshold, consistent with Classical Test Theory guidelines (Siegert et al., 2022). These findings align with the theoretical premise that well-constructed latent variables yield reliable and interpretable psychometric outcomes. This supports the robustness of organizational health assessment through validated instruments such as the OHBI. Inter-item correlation analysis (Table 7) further demonstrated strong internal consistency within constructs and acceptable discriminant validity between constructs, consistent with the multi-trait multi-method (MTMM) framework for construct distinctiveness (Campbell and Fiske, 1959).

Confirmatory factor analysis further validated the measurement model (Figure 2), with strong model fit indices (CFI = 0.978; RMSEA = 0.048; Table 9) and all factor loadings exceeding 0.70 (Table 10). The constructs also demonstrated strong convergent and discriminant validity (Tables 11–13). Collectively, these psychometric findings affirm the theoretical soundness of the OHBI as a diagnostic tool grounded in measurement theory and organizational behavior research. The validation of awareness, appreciation, and communication satisfaction as core dimensions of organizational health strengthens the empirical foundation of OHBI and reinforces its relevance in culturally specific contexts such as Saudi Arabia (Healthy Workplace, 2025).

Organizational health is increasingly recognized as a system-level capability that enables organizations to adapt, align, and sustain performance amid internal and external challenges (Xenidis and Theocharous, 2014; Soehartono et al., 2023). As Doganay and Dagli (2020) argue, a healthy organization is not only efficient but also capable of continuous learning and renewal. In this context, the present findings extend existing literature by confirming that awareness, appreciation, and communication satisfaction each significantly contribute to employee engagement, both independently and through interaction effects, as shown in Table 14. The structural model demonstrated excellent fit (χ^2^/df = 3.1; CFI = 0.994; TLI = 0.988; RMSEA = 0.025), indicating that the model effectively captures the relationships among the study constructs. Addressing the research questions derived from identified gaps in prior literature, the study yielded several key findings that advance understanding of organizational health and employee engagement within the Saudi Arabian context.

*RQ1*: To what extent does awareness significantly predict employee engagement within the context of Saudi organizations?

The findings indicate a strong and statistically significant relationship between awareness and employee engagement (*β* = 0.230, *p* < 0.001), confirming Hypothesis H1 and supporting RQ1. Awareness—defined as employees’ understanding of their organization’s mission, vision, policies, and internal communications—emerged as the strongest predictor of engagement. This finding aligns with Organizational Transparency Theory, which posits that open communication regarding vision, strategy, and expectations enhances trust and motivation (Albu and Flyverbom, 2019). Prior research further supports that employees equipped with strategic knowledge are more likely to engage proactively and contribute meaningfully to organizational outcomes (Urbański, 2021; Al-Dmour et al., 2020). This relationship is particularly salient in the Saudi context, where strategic alignment is central to the successful realization of Vision 2030.

*RQ2*: How does appreciation contribute to the variance in employee engagement across diverse institutional sectors?

The second research question is addressed by the positive and significant association between appreciation and employee engagement (*β* = 0.152, *p* < 0.001), supporting H2 and RQ2. Appreciation, operationalized as consistent recognition of employees’ contributions, demonstrated a robust influence across sectors. This finding is consistent with prior studies showing that both tangible rewards and intangible recognition enhance employee morale and commitment (Sadilla and Wahyuningtyas, 2023). From a Self-Determination Theory (SDT) perspective, intrinsic motivation, autonomy, and perceived competence are central drivers of engagement (Chiu, 2022). Ryan and Deci (2020) further emphasized that future SDT research should focus on motivational design mechanisms that enhance engagement and learning. Accordingly, recognition—even in small forms—serves as a powerful signal of value and belonging within the organization.

*RQ3*: What is the direct association of communication satisfaction on employee engagement, and to what degree does it serve as an independent predictor?

Communication satisfaction (*β* = 0.070, *p* < 0.001), although a comparatively smaller predictor than awareness or appreciation, demonstrated a statistically significant independent association with employee engagement, supporting H3 and addressing RQ3. This finding reinforces prior evidence that communication satisfaction functions as a direct antecedent of engagement by shaping employees’ perceptions of inclusion, clarity, and organizational support (Špoljarić and Tkalac Verčič, 2022; Lin and Huang, 2021).

*RQ4*: Does communication satisfaction significantly moderate the relationship between awareness and employee engagement, thereby amplifying or attenuating this association?

The positive and statistically significant interaction between awareness and communication satisfaction (*β* = 0.082, *p* < 0.001) supports H4 and answers RQ4. This result indicates that communication satisfaction strengthens the positive relationship between awareness and engagement, such that awareness contributes more strongly to engagement when communication is perceived as effective. This finding is consistent with Communication Climate Theory, which emphasizes that supportive communication environments enhance psychological safety and employee involvement (Men and Bowen, 2021). When communication is timely, clear, and trusted, employees are more likely to internalize organizational goals and translate awareness into active engagement. From an HR perspective, establishing feedback loops, listening mechanisms, and effective message cascading is critical in helping employees understand the rationale behind strategic initiatives, thereby fostering commitment and participation (Von Moltke, 2025).

*RQ5*: Does communication satisfaction exert a moderating influence on the association between appreciation and employee engagement, and if so, in what direction?

A notable finding of the present study is the negative moderating effect of communication satisfaction on the relationship between appreciation and employee engagement. Although appreciation demonstrated a positive main effect on engagement, its influence weakened at higher levels of communication satisfaction (*β* = −0.027). This pattern suggests that appreciation and communication satisfaction do not function as purely additive motivational resources. Drawing on the Job Demands–Resources (JD-R) model, the logic of resource substitution suggests that when communication resources are abundant and of high quality, employees may become less reliant on interpersonal recognition as a primary source of motivation (Bakker and Demerouti, 2017).

In addition, communication overload theory proposes that excessive communication—even when well intentioned—can increase cognitive load, dilute emotional signals, and reduce the motivational impact of recognition (Eppler and Mengis, 2004). From a “too-much-of-a-good-thing” perspective, once communication satisfaction reaches a saturation point, the marginal motivational value of additional appreciation diminishes, resulting in attenuated engagement effects (Grant and Schwartz, 2011). Importantly, this negative interaction does not contradict appreciation theory or intrinsic motivation principles (Deci et al., 1999) but instead reflects a resource-balancing mechanism within organizational health systems. Appreciation appears to contribute most strongly to engagement when communication resources are present at moderate rather than excessive levels.

This study provides crucial insights into how key components of organizational health—specifically awareness, appreciation, and communication satisfaction—interact to influence employee engagement in Saudi Arabian organizations. Drawing on data from 7,548 employees across diverse regions and industries, the findings highlight the importance of adopting a multidimensional framework, such as the Organizational Health Behavior Index (OHBI), to enhance employee engagement in alignment with the national transformation objectives of Vision 2030 (AlRuthia et al., 2018). Theoretically, the results support the position that the OHBI functions as a robust diagnostic model for identifying organizational health dimensions that are statistically associated with employee engagement through both direct and interaction effects. The scale’s multifaceted design, incorporating strategic and behavioral dimensions, enables a comprehensive understanding of the mechanisms linking organizational health and employee engagement.

From a practical perspective, the findings suggest that HR leaders should design interventions that simultaneously strengthen employee awareness, reinforce appreciation practices, and develop effective communication satisfaction networks. Optimizing communication satisfaction should not be viewed merely as a support function but as a strategic lever that amplifies the motivational impact of other engagement drivers. Contemporary performance management systems increasingly emphasize continuous feedback, coaching, recognition, and digital enablement, all of which can leverage OHBI insights by fostering communication environments that enhance awareness and appreciation (Barry et al., 2020). Such alignment not only increases employee engagement but also contributes to healthier organizational functioning.

Finally, the study carries important policy implications. In the Saudi Arabian context—where public and private sector reform and workforce nationalization remain key priorities—enhancing engagement through healthy organizational practices can act as a catalyst for sustainable transformation. Prior research emphasizes that organizational health is closely linked to long-term success, particularly during periods of change and uncertainty (Barry et al., 2020). Accordingly, embedding awareness, appreciation, and communication satisfaction into HR strategies may yield substantial gains in employee creativity, retention, and productivity.

### 5.1 Theoretical implication

From a theoretical standpoint, this study contributes to the expanding discourse on organizational health by validating the Organizational Health Behavior Index (OHBI) as a multidimensional diagnostic framework for understanding employee engagement. Beyond this, integrating the OHBI within established theoretical models—such as the Job Demands–Resources (JD-R) model, which explains how organizational environments shape employee well-being and performance (Tummers and Bakker, 2021), and the Ability–Motivation–Opportunity (AMO) framework, which links HRM practices to employees’ ability, motivation, and opportunity to perform (Kellner et al., 2019)—can further strengthen its theoretical grounding.

Within the JD-R framework, OHBI dimensions—awareness, appreciation, and communication satisfaction—can be conceptualized as critical organizational resources that help buffer job demands and promote employee engagement. Similarly, within the AMO framework, awareness may correspond to the “ability” to align with organizational goals, appreciation may function as a “motivation” enhancer, and communication satisfaction may represent the “opportunity” for meaningful participation. Framing OHBI through these complementary theoretical lenses provides a more comprehensive explanation of how organizational health behaviors translate into sustained employee engagement and performance outcomes.

### 5.2 Future implications and limitations

The findings of this study position the Organizational Health Behavior Index (OHBI) as a promising diagnostic framework for evaluating employee engagement through key behavioral drivers such as awareness, appreciation, and communication satisfaction. Although the current study is situated within the Saudi Arabian context, the core dimensions of the OHBI are grounded in universal organizational dynamics—clarity of purpose, recognition, and effective internal communication—that are relevant across cultures and sectors. This suggests strong potential for the adaptation and application of the OHBI in diverse cultural and national settings, particularly when tailored to reflect local organizational norms and values. Future cross-cultural validation studies may further enhance its global relevance and generalizability.

Another limitation relates to the gender imbalance in the sample, as 85.2% of participants were male. While this distribution reflects the workforce composition of several Vision 2030–targeted sectors included in the study, it limits the generalizability of the findings across gender. Multi-group analysis would have been informative; however, the relatively small number of female respondents did not meet the statistical requirements for reliable multi-group CFA or SEM, and such analyses could have produced unstable results. Future research should therefore adopt more gender-balanced or stratified sampling strategies to enable robust examination of gender-based differences in organizational health and employee engagement.

A further limitation stems from the context-specific and cross-sectional nature of the study, which constrains causal inference and broader transferability. Longitudinal and cross-national research designs are recommended to strengthen the cross-contextual robustness and predictive validity of the OHBI.

## 6 Conclusion

This research study advances to the understanding of the collective and interactive influence of organizational health dimensions—namely awareness, appreciation, and communication satisfaction—on employee engagement in Saudi Arabia. The study applied the Organizational Health Behavior Index (OHBI) to a diversified population, providing insights into their interplay and confirming that these characteristics strongly predict employee engagement which is an essential dimension of healthy workplace. The results emphasized that important open communication and acknowledgment are for improving motivation and alignment, particularly in the Saudi Arabian sociocultural setting and its Vision 2030 reform plan. HR is vital in crafting clear, consistent communication that helps employees understand the “why” behind strategic changes. Establishing feedback mechanisms, addressing issues, and assisting managers in disseminating critical messages are imperative in this context. Engaged personnel are far more inclined to endorse and facilitate the achievement of strategic projects. This research provides theoretical insights and practical guidance for HR professionals aiming to cultivate healthier, more engaging, and high-performing workplaces by validating OHBI as a multidimensional diagnostic instrument. The OHBI can foster a workplace environment that enhances productivity, engagement, and retention. Organizations can evaluate their health behavior ratings against industry standards or best practices with the study model.

## Data Availability

The original contributions presented in the study are included in the article/Supplementary material, further inquiries can be directed to the corresponding author.
