# Correction: The role of awareness, appreciation, and communication satisfaction in shaping employee engagement: evidence from the organizational health behavior index

**DOI:** 10.3389/fpsyg.2026.1922698

**Published:** 2026-07-10

**Authors:** Abad Alzuman, Muath Jaafari, Zaiba Ali, Rahila Ali, Lina Ibrahim Bakadam

**Affiliations:** 1 Department of Management, College of Business Administration, Princess Nourah Bint Abdulrahman University, Riyadh, Saudi Arabia; 2 Organizational Culture and Internal Communication, ICM, Amjad Watan, Riyadh, Saudi Arabia; 3 Faculty of Management, Jagran Lakecity University, Bhopal, India

**Keywords:** appreciation, awareness, communication satisfaction, employee engagement, moderation, organizational health behavior index (OHBI), Saudi Arabia, SEM

In the published article an incorrect Funding statement was provided as This study was supported by internal research funding from Princess Norah University, Riyadh, SA, which provided institutional support for data collection and analysis.

The correct funding statement reads:

