# Supplementary material for: The role of awareness, appreciation, and communication satisfaction in shaping employee engagement: evidence from the organizational health behavior index
Source: Front Psychol. 2026 Feb 9;17:1671007. doi: 10.3389/fpsyg.2026.1671007 (PMC12920602; doi:10.3389/fpsyg.2026.1671007)
Supplement: Supplementary file 1 [file Data_Sheet_1.docx]

| S.no | Items | Theme |
| --- | --- | --- |
| 1 | I am aware of my organization's vision, mission, and goals. | Awareness |
| 2 | I am implementing to my organization values in my daily work. | Awareness |
| 3 | I am aware of my organization external and internal news. | Awareness |
| 4 | I am aware of my rights and privileges as an employee in my organization | Awareness |
| 5 | The appreciation my line manager shows for my work and accomplishments is satisfactory to me. | Appreciation |
| 6 | My company supports my efforts by providing the resources required to accomplish my tasks more effectively. | Appreciation |
| 7 | I rarely think about leaving my organization to work someplace else | Appreciation |
| 8 | I maintain a very strong relationship with colleagues in other departments. | Relations |
| 9 | My relationship with my line manager is perfect. | Relations |
| 10 | My family has a good relationship with my organization (they are aware of my work  responsibilities, value the services offered by the organization, and stay informed about its news). | Relations |
| 11 | I am keen on promoting my organization’s accomplishments and activities, both on my  personal social media profiles and during my social engagements. | Engagement |
| 12 | During work hours, time flies by without me noticing as I focus on my tasks and responsibilities. | Engagement |
| 13 | I am familiar with the professional backgrounds of my colleagues in the same department, their health conditions, and the best ways to interact with them in general. | Engagement |
| 14 | I would recommend my organization as a great place to work for my colleagues and family. | Engagement |
| 15 | My organization interacts positively with global awareness days, standing out compared to other organizations. | Comm satisfaction |
| 16 | I am satisfied with the communication services offered by my organization last year,  including email communications, motivational programs, and recreational and interactive activities for employees. | Comm satisfaction |
| 17 | Internal communication was able to reach me through various communication channels. | Comm satisfaction |
| 18 | In your opinion, what priorities does your organization focus on the most?  A. Employees B. Projects or Customers C. Products or Innovation D. Quality and System | Culture |
| 19 | Choose the most appropriate behavior that describes your line manager:  A. Supportive - Mentor B. Inspiring - Risktaker  C. Productive - Hardworking D. Cautious – Adheres to rules | Culture |
| 20 | Choose the most appropriate behavior that describes your colleagues:  A. Supportive B. Productive C. Creative D. Cautious - Rule-followers | Culture |
| 21 | Identify the top three personal interests that appeal to you :  A. Sports and Health B. Technology and Gaming C. Entertainment and Shopping D. Finance and Business E. Restaurants and Cafés F. Education and Development G. Volunteering and Community Service H. Travel and Tourism I. Wildlife and Nature J. Culture and Arts | Persona |
| 22 | What are the key aspects you know about your colleagues:  A. Their health condition B. Their hobbies and talents C. Their professional and academic history D. How to deal with them F. General information about them E. Not interested | Persona |
| 23 | The top four ways that represent appreciation to you are:  A. Administrative awards B. Financial compensation C. Highlighting and showcasing my achievements D. Hearing my opinions and including me in key decisions E. Focusing on my mental well-being F. Investing in my professional growth and career G. Verbal appreciation with expressions of thanks H. Material gifts I. Clear information about my rights and benefits J. Financial perks | Persona |
| 24 | Best three communication channels to deliver messages for you:  A. E-mail B. Intranet / Internal Portal C. Snapchat D. Display Screens E. Telegram F. Text Messages (SMS) G. Whatsapp H. Wall stickers I. Workshops | Persona |
| 25 | To whom do you feel the greatest sense of appreciation?  A. Your Colleagues B. Your Manager C. Your Organization | Persona |
| 26 | In your opinion, what are the key aspects of our work environment that need improvement and development? | Open-ended |
| 27 | In your opinion, what are the key positive aspects of our work environment that we should strive to reinforce? | Open-ended |
